# Lineage relationship of prostate cancer cell types based on gene expression

**DOI:** 10.1186/1755-8794-4-46

**Published:** 2011-05-23

**Authors:** Laura E Pascal, Ricardo ZN Vêncio, Robert L Vessella, Carol B Ware, Eneida F Vêncio, Gareth Denyer, Alvin Y Liu

**Affiliations:** 1 Department of Urology University of Washington, Seattle, WA 98195, USA; 2 Institute for Stem Cell and Regenerative Medicine, University of Washington, Seattle, WA 98195, USA; 3 University of Pittsburgh Medical Center, Department of Urology, Pittsburgh, PA 15232, USA; 4 Genetics Department, University of São Paulo's Medical School at Ribeirão Preto, Brazil; 5 Department of Comparative Medicine, University of Washington, Seattle, WA 98195, USA; 6 Department of Pathology, Federal University of Goias, Goiania, Brazil; 7 Department of Biochemistry, The University of Sydney, Sydney, Australia

## Abstract

**Background:**

Prostate tumor heterogeneity is a major factor in disease management. Heterogeneity could be due to multiple cancer cell types with distinct gene expression. Of clinical importance is the so-called cancer stem cell type. Cell type-specific transcriptomes are used to examine lineage relationship among cancer cell types and their expression similarity to normal cell types including stem/progenitor cells.

**Methods:**

Transcriptomes were determined by Affymetrix DNA array analysis for the following cell types. Putative prostate progenitor cell populations were characterized and isolated by expression of the membrane transporter ABCG2. Stem cells were represented by embryonic stem and embryonal carcinoma cells. The cancer cell types were Gleason pattern 3 (glandular histomorphology) and pattern 4 (aglandular) sorted from primary tumors, cultured prostate cancer cell lines originally established from metastatic lesions, xenografts LuCaP 35 (adenocarcinoma phenotype) and LuCaP 49 (neuroendocrine/small cell carcinoma) grown in mice. No detectable gene expression differences were detected among serial passages of the LuCaP xenografts.

**Results:**

Based on transcriptomes, the different cancer cell types could be clustered into a luminal-like grouping and a non-luminal-like (also not basal-like) grouping. The non-luminal-like types showed expression more similar to that of stem/progenitor cells than the luminal-like types. However, none showed expression of stem cell genes known to maintain stemness.

**Conclusions:**

Non-luminal-like types are all representatives of aggressive disease, and this could be attributed to the similarity in overall gene expression to stem and progenitor cell types.

## Background

Tumor heterogeneity is a major hurdle in effective treatment of the disease. This heterogeneity could be due to multiple cancer cell types with distinct gene expression. How do these cell types arise? The cancer stem cell hypothesis states that tumors are propagated by cancer cells with stem-cell characteristics, and that tumor heterogeneity results from differentiation of these stem-like cells. Tumors from several tissue types have been found to contain specific populations of tumorigenic and non-tumorigenic cells. Breast tumor formation can be initiated by a small number of tumorigenic cells characterized as CD44^+^CD24^lo/-^, while non-tumorigenic cells are CD44^-^CD24^+^. The latter could be generated from the former during tumor growth [1]. Tumorigenicity is assayed by xenograft implantation and tumor expansion in immune-compromised hosts. In leukemia, tumorigenic cells share a phenotype of CD34^+^CD38^- ^with normal hematopoietic stem cells [2]. Tumorigenic or cancer stem cells that are typed CD133^+^CD44^+^CD49b^+^CD29^+ ^have also been reported for prostate tumors [3]. To date, these cluster designation (CD) cell surface molecules are the principal markers used to qualify these tumorigenic cells as cancer stem cells, and the fact these cells can apparently undergo differentiation to produce other types.

Prostate cancer is a common cancer in men in the Western countries, and the second leading cause of cancer mortality [4]. Why the human prostate is prone to developing cancer and what the molecular mechanism of the disease process remain unanswered. In prostate development, epithelial differentiation is mediated by stromal mesenchyme induction of stem cells [5]. Thus, epithelial elements containing stem/progenitor cells isolated from either the prostate or the bladder can be induced by prostatic stromal cells to produce only prostate-like structures [6]. Presumably, bladder stromal cells would induce bladder-like structures instead if that experiment was done. This induction could be defective in cancer due to abnormal gene expression by the tumor-associated stromal cells [7]. The lack of appropriate stromal signaling may lead to abnormal epithelial differentiation giving rise to diseases like cancer. The alternative is that a cancer stem cell emerges after accumulating enough critical somatic DNA mutations over time, and this then differentiates into cancer epithelial cells (and perhaps the cancer-associated stromal cells as well).

In this report, we used cell type-specific transcriptomes obtained in our lab to examine possible lineage relationship between prostate cancer cell types and normal cell types including that of stem/progenitor. Our goal was to determine the extent of stem-cell gene expression not only of the CD molecules but also of all others in cancer, and to see how this gene expression was correlated with tumor biology. The cancer cell types included prostate cancer cell lines LNCaP, C4-2, CL1, PC3, DU145, tumor xenografts LuCaP 35 and LuCaP 49, CD26^+ ^Gleason pattern 3 (G3) and pattern 4 (G4) cancer cells isolated from primary tumors [8]. G3 cancer cells are typical of well-differentiated tumors showing glandular histoarchitecture, while G4 cancer cells are of tumors without glandular differentiation [9]. Tumors with a significant component of G4 are associated with poor outcome. The in vitro cultured cell lines were established from metastasis: lymph node for LNCaP, bone for PC3 and brain for DU145 [10]. C4-2 and CL1 were derived from LNCaP through selection in castrated animals and androgen-depleted growth media, respectively. The in vivo maintained LuCaP 35 was derived from a lymph node metastasis and shows features of a prostate-specific antigen (PSA/KLK3)-producing adenocarcinoma [11]. LuCaP 49 was established from an omentum metastasis and shows features of a PSA non-producing neuroendocrine small cell carcinoma [12]. Thus, a wide spectrum of this disease is covered by these different cell types. In this analysis, stem cell types were represented by the embryonal carcinoma (EC) cell line NCCIT (hyperdiploid, established from a nonseminomatous germ cell tumor) [13], and embryonic stem (ES) cell line H1 (WA01, karyotype 46, XY) [14]. EC and ES cells have been shown to have very similar gene expression [15], and are stained positive for stem cell-specific alkaline phosphatase. We have reported that NCCIT could undergo differentiation with loss of stem cell markers, growth retardation, and altered morphology under the influence of either prostate or bladder stromal cells in co-culture [16]. Non-cancer cell types were represented by sorted CD26^+ ^luminal epithelial, CD104^+ ^basal epithelial, CD49a^+ ^stromal smooth muscle, CD31^+ ^endothelial cells [17], and prostate cell populations identified and isolated by their expression of the transmembrane ATP-binding cassette transporter ABCG2 [18]. Isolation of ABCG2^+ ^cells was done either by the use of anti-ABCG2/CDw338 monoclonal in magnetic cell sorting (MACS) or via flow cytometry of side populations. Side populations are due to the ability of ABCG2 to efflux a DNA dye (which ABCG2-negative cells in the main population cannot), and bone marrow side populations are enriched for marrow graft repopulating stem cells. The ABCG2^+ ^cells were used to represent adult organ progenitor cells of the prostate whereas ES and EC cells were of germ cells. All transcriptome datasets are available on our public database UESC [19], which is also incorporated in the NCI-EDRN (Early Detection Research Network) website. Data quality of Affymetrix-derived transcriptomes was assessed for correspondence between array hybridization signals of CD genes and CD immunostaining results [20]. In general, there was a good fit between the two data types, i.e., immunostained cell types showed array hybridization signals for the corresponding CD genes. For lineage relationship, we employed a principal components analysis of array datasets to characterize the relatedness between cell types as defined by their transcriptomes, i.e., the entire repertoire of expressed genes.

## Materials and methods

### Prostate cancer cell lines and xenografts

Prostate cancer cell lines CL1 (and its subclones CL1.1 and CL1.31) [21,22], DU145 and PC3 were cultured and harvested for array analysis as described in our previous report on differential gene expression between LNCaP and C4-2 [23]. The LuCaP series of xenografts were generated in our department from surgical specimens and donor necropsies implanted subcutaneously in mice [24]. All specimens were obtained after informed consent and collected using protocols approved by the Institutional Review Board and Human Subject Division at the University of Washington. The tumors were serially passaged in mice and harvested for analysis. Samples of LuCaP 35 and LuCaP 49 at five different passages were analyzed by DNA arrays to examine if gene expression was stable over time. NCCIT cells were cultured in RPMI1640 media [16], and H1 cells at passage 70 were cultured as previously described [14] either with mouse embryonic fibroblast (MEF) feeder or in MEF-conditioned media, and for the last two passages before harvest in TeSR2 media (STEMCELL Technologies, Vancouver, Canada) on growth factor-reduced Matrigel (BD Biosciences, San Jose, CA).

### DNA array analysis

Quality and concentration of RNA prepared from cells or xenografts were determined by Agilent 2100 Bioanalyzer and RNA Labchip (Agilent Technologies, Santa Clara, CA). Human Genome U133 Plus 2.0 GeneChips (Affymetrix, Santa Clara, CA) were used for expression profiling. The U133 array contained probesets representing 54,675 genes, splice variants, and ESTs. The GeneChips were prepared, hybridized, and scanned according to the protocols provided by Affymetrix (P/N 702232 Rev. 2) [23]. RNA prepared from LuCaP 35, LuCaP 49 and H1 were reverse transcribed with poly (dT)/T7 promoter primer, and the cDNA was made double-stranded. In vitro transcription was performed with biotinylated ribonucleotides, and the biotin-labeled cRNA was hybridized to the GeneChips. The chips were washed and stained with streptavidin-PE using FS-450 fluidics station (Affymetrix). Data was collected with Affymetrix GeneChip Scanner 3000. All five samplings of each xenograft at different passages were analyzed.

### Prostate cell type-specific transcriptomes

Previously determined cell type-specific transcriptomes were downloaded from UESC [19]. The sorted cell populations were isolated from prostate tissue specimens obtained from patients undergoing radical prostatectomy [8,17,18]. Both cancer and non-cancer samples were digested by collagenase for cell isolation by MACS. Transcriptomes were obtained for CD104^+ ^basal, CD26^+ ^luminal, CD49a^+ ^stromal, CD31^+ ^endothelial, ABCG2^+ ^(5D3) or side-population (SP) progenitor, CD26^+ ^cancer cells of Gleason 3+3 (G3) and 4+4 (G4) primary tumors. Transcriptomes were also available for the prostate cancer cell lines LNCaP, C4-2, CL1, DU145, PC3 as well as those of NCCIT and H1.

### Computational analysis of datasets

For differential gene expression, datasets were analyzed by HTself, a self-self based statistical method for low replication microarray data, specifically those obtained from isolated cancer cell types [8]. To apply this method, all possible combination of pair-wise comparisons among experiments were taken to create sets of ratios. Gene expression level was defined as the normalized and summarized intensities of each GeneChip probeset, and was presented as its logarithmic value: *X *= log_2_(Normalized intensity). This step was carried out using the standard robust multi-array average (RMA) method [25], implemented in the analysis pipeline SBEAMS [26]. The strength of differential expression between any pair of experiments was estimated by *M*_*i *_= log_2_(ratio) = *X*_*i*_-*X*_*a*_, where *a *represented one particular cell type and *i *represented each given cell type in the set. A probeset was considered significantly differentially expressed if at least 80% of its log-ratio combinations were outside the 99.9% credibility intensity-dependent cutoff. Moreover, an average greater than 8-fold difference in expression level was chosen.

In principal components analysis (PCA) of the transcriptome datasets, a gene expression subspace was obtained that highlighted the principal sources of variability among transcriptomes of the different prostate cell types. This space was created with transcriptomes of the four cell types isolated from the prostate: luminal L, basal B, stromal S and endothelial E. A rotation matrix was obtained by using averages of *X*_*L*_, *X*_*B*_, *X*_*S*_, and *X*_*E*_, and these were plotted as projections on the principal components graph (see Supplemental File 1, http://labpib.openwetware.org/PCA.html). The other transcriptomes were then projected into this PCA-generated subspace, which could be rotated freely to visualize spatial separation of the individual datapoints denoting the cell types. The following datasets were thus analyzed: CD26^+ ^luminal, CD49a^+ ^stromal, CD104^+ ^basal, CD31^+ ^endothelial, ABCG2^+ ^progenitor (5D3), SP, CD26^+ ^G3 cancer, CD26^+ ^G4 cancer, LNCaP, C4-2, CL1 (and subclones), DU145, PC3, LuCaP35, LuCaP49, NCCIT and H1.

## Results

### Relatedness between prostate progenitor cells and stem cells

**Figure 1:**
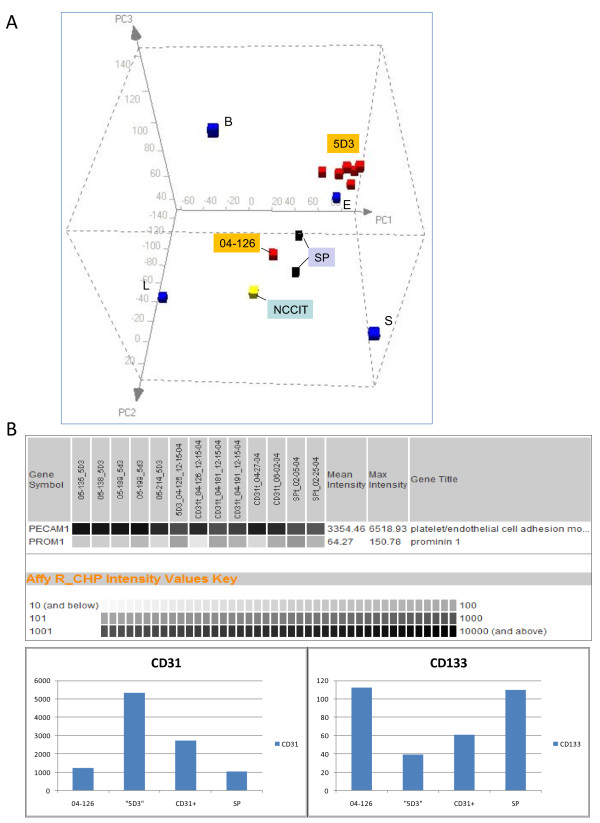
**Principal components analysis of prostate ABCG2**^**+ **^**cells**. A: This human prostate PCA space is keyed on the transcriptomes of B, L, S, and E cells. Stem cell transcriptome is represented by that of NCCIT. Putative progenitor cells sorted from the prostate by antibody to ABGC2 (clone 5D3) or in SP are shown. Many 5D3 sorts (red cubes) are found to cluster near E except for that derived from specimen 04-126. This and two SPs are positioned nearer the NCCIT. B: The expression levels of CD133 and CD31 in 04-126, other 5D3 sorts (labeled as "5D3"), endothelial sorts (labeled as CD31+) and SP are indicated by array signal values on a gray scale in the data query display. The histograms below show the same query output. Note the different expression magnitude scales on the *y*-axis for the two genes.

The prostate SP was isolated due to its slower uptake of Hoechst 33342 due to ABCG2. SP was collected from cells pre-selected by MACS using anti-CD44 since ABCG2^+ ^cells were detected in the CD44^+ ^basal epithelium of the prostate. They constituted a minor population (< 1%) and differed from ABGC2^+ ^endothelial cells of capillaries by expression of the basal marker CD138/SDC1 [18]. In addition to SP, the ABCG2^+ ^cells were sorted from sizable benign tissue specimens using the ABCG2 antibody clone 5D3, and labeled as 5D3 populations [18]. Figure 1A shows a PCA subspace defined by the transcriptomes of basal, luminal, stromal, and endothelial. These four differentiated cell types are distinct in gene expression, and are widely separated in this three dimensional plot. The distance between any two datapoints is a measure of the extent of differential gene expression. Transcriptomes of the SP and 5D3 cells were then projected into this "human prostate" PCA subspace plus the transcriptome of NCCIT used as that of human pluripotent stem cells.

First, a majority of 5D3 cell sorts were clustering near the endothelial cells. In our sorting scheme, CD31/PECAM1 antibody was first used as a means to purge CD31^+^ABCG2^+ ^endothelial cells in the tissue digests before sorting by the 5D3 antibody. However, significant amounts of any residual endothelial cells in the resultant, presumably CD31^-^ABCG2^+^, populations would skew the expression profile towards E given the rarity of progenitor cells. Nevertheless, one particular 5D3 population obtained from specimen 04-126, plotted closer to NCCIT apart from the other 5D3 sorts, might represent a progenitor population with less endothelial contamination. The array signal intensity value for the CD31 gene in 04-126 was 1235 compared to 5336 (average of five) for the other 5D3 populations, 2719 (average of five) for the CD31-sorted endothelial populations, and 1049 (average of two) for SP. The signal value for CD133 in 04-126 was 113 compared to 40 for the other 5D3 sorts and 61 for the CD31 sorts, and 110 for SP (Figure 1B). For the expression of these two markers, the 5D3 of 04-126 and SP were similar, and both populations (isolated by different methodologies) were found plotted near the EC cells. Some of the differences could be attributed to genes involved in cell proliferation since the EC cells were harvested from in vitro cultures. Second, although ABCG2^+ ^cells and basal cells share CD specificities (e.g., CD44, CD49f, CD138), the 04-126/SP cluster was distal from the ABCG2^- ^basal B signifying that the overall gene expression was significantly different between these cell types. Based on this transcriptome analysis, basal cells did not appear to represent a possible progenitor population of secretory cells, which was postulated by some investigators (see ref. [27]), by showing less gene expression overlap with EC cells than the ABCG2^+ ^cells.

**Figure 2:**
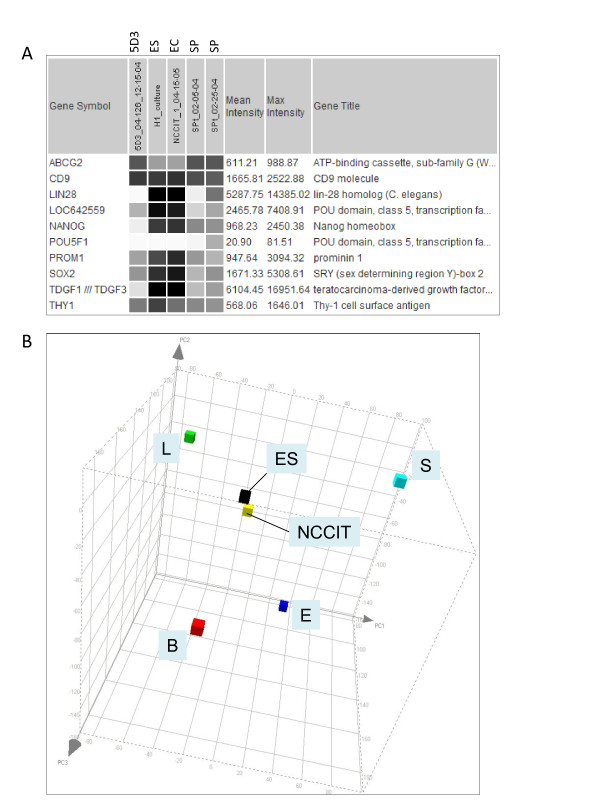
**Gene expression comparison of ABCG2**^**+ **^**cells and stem cells**. A: Differential expression of selected genes between 5D3 (04-126), SP and ES (H1) and EC (NCCIT) cells as obtained from dataset query is shown. B: PCA plot of H1 and NCCIT shows the close relatedness between these two cell types.

Dataset comparison was carried out between SP, 5D3 (04-126) and EC, ES. The expression pattern of several genes in these populations is shown in Figure 2A. First, note the similarity between EC and ES cells in the expression levels of stem cell markers SOX2, LIN28, NANOG, POU5F1/LOC642559, TDGF1 (CRIPTO) and PROM1 (CD133). In addition, stem cell marker could also be considered for CD9 [28] and THY1 (CD90) [29]. Figure 2B shows the proximity of ES (H1) and EC (NCCIT) in gene expression by PCA. For POU5F1 (OCT4), two Affymetrix probesets (235842_at, 238997_at) produced no signal while two others, LOC642559 (210265_x_at) and LOC645682 (210905_x_at), gave strong signals. Both SP and 5D3 cells showed low expression levels of these stem cell genes. NCCIT was negative for ABCG2 expression [18], so were the ES cells in contrast to SP and 5D3, which were obtained based on ABCG2 expression. Despite these differences, the overall gene expression of the 5D3 and SP populations was more similar to that of stem cells than to any of the differentiated cell types (i.e., L, S, B, E) as indicated by PCA. There were 13,413 genes with signal intensity values > 100 expressed in common by all three (> 80%); 15,899 between ES and EC; 14,404 between ES and 5D3; 14,913 between EC and 5D3. The common pool for SP and ES/EC was 13,122, and 16,300 between 5D3 and SP. Accordingly, the PCA display could be viewed as a cell differentiation space in which the undifferentiated stem/progenitor cells appeared to occupy a more interior position and the differentiated cell types were positioned towards the periphery. Relatedness between cell types was indicated by the separation of the datapoints. Thus, the gene expression difference between the epithelial cell types (luminal and basal) was almost as large as that between epithelial and stromal cells. These three prostate cell types have their individual complement of CD molecules [30].

### 2. Prostate cancer cell types as defined by transcriptome

**Figure 3:**
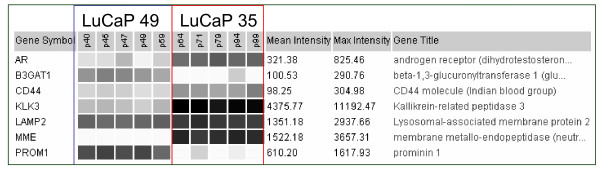
**LuCaP xenograft gene expression**. Displayed are the expression patterns of selected genes (listed in the first column) in the two LuCaP xenografts with individual datasets identified by their passage numbers p.

The cancer cell types analyzed were cells sorted from tumor tissue specimens, cancer cell lines cultured in vitro, and xenografts grown in vivo. Array results for the LuCaP xenografts at various passages showed minimal gene expression changes during serial transplantation in mice, from p64 to p99 for LuCaP 35 and p40 to p59 for LuCaP 49. There was no evidence in enrichment of CD stem cell markers over time, e.g., CD44 in LuCaP 35 (Figure 3). These tumor cells appeared to maintain their expression profile during long-term growth as indicated by the relative array signal levels of the genes in Figure 3. Dataset query confirmed that LuCaP 49 was negative for androgen receptor (AR), KLK3, whereas LuCaP 35 was positive for these two markers. Furthermore, data query showed that LuCaP 49 was CD57^+ ^(B3GAT1; a marker associated with cells showing neuroendocrine differentiation), CD44^-^, CD107b^+ ^(LAMP2, expressed by many prostate cancer cell types [31]), CD10^-^, CD133^+^; LuCaP 35 was CD57^-^, CD44^-^, CD107b^+^, CD10^+^, CD133^-^. These CD reactivities had been verified by flow cytometry analysis and immunostaining [12,32].

**Figure 4:**
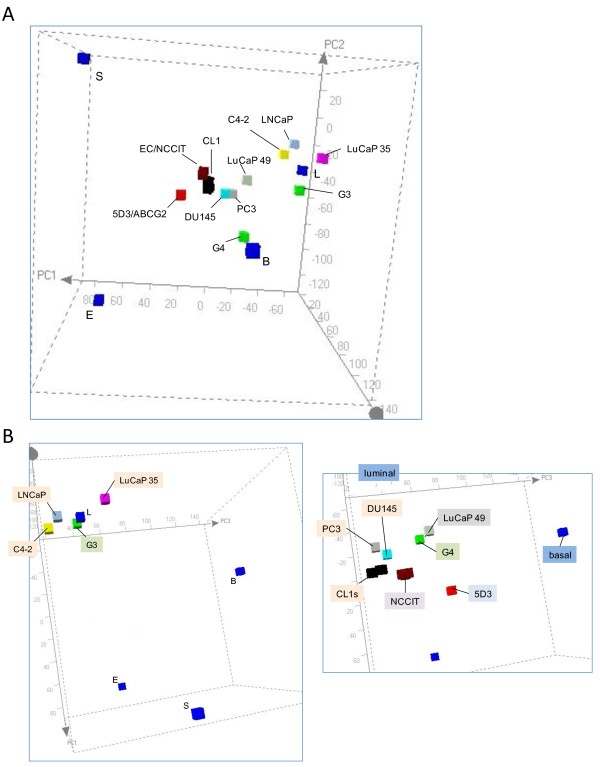
**PCA of prostate cancer cell types**. A: Transcriptomes of prostate primary cancer G3, G4, cell lines LNCaP, C4-2, CL1, DU145, PC3, xenografts LuCaP 35, LuCaP 49 are projected into the space defined by B-L-S-E. The CL1 data point also contains transcriptomes from CL1 subclones. B: Panel A shows grouping of the so-called luminal-like cancer cell types, while panel B shows grouping of the non-luminal-like cancer cell types in a different viewing aspect.

The PCA plot shown in Figure 4A incorporated the transcriptomes of LNCaP, C4-2, CL1, PC3, DU145, LuCaP 35, LuCaP 49, G3 cancer (specimen 05-179) and G4 cancer (specimen 08-032), plus NCCIT as a cancer stem cell type. For the cell lines, C4-2 was derived from LNCaP, and their transcriptomes have been shown by us to be very similar with 90% of the genes detected [23], and this was indicated by PCA. One notable difference was CD26 (Table 1). A small CD26^+ ^population could be detected and expanded after CD26 sorting from overall CD26^- ^LNCaP cultures. This subpopulation could be related to the CD26^+ ^C4-2 [32]. This result suggested that CD subpopulations could also exist in the LuCaP tumors and others. CL1 was also derived from LNCaP, and several clones (e.g., CL1.1, CL1.31) were further obtained showing different growth characteristics in vivo and in vitro. The CL1s were plotted in the vicinity of PC3 and DU145. Therefore, these three cell lines were scored very similar in overall gene expression, which was supported by their CD phenotype (Table 1). The table shows that none of the cancer cell types listed was positive for both CD44 and CD133, markers most often cited as associated with cancer stem cells. The CD44^-^CD133^- ^LuCaP 35 could be propagated in mice equally successfully as the CD44^-^CD133^+ ^LuCaP 49, although whether small numbers of these tumor cells could form tumors has not been tested. LuCaP 35, like LNCaP, was derived from a lymph node metastasis. Thus, not surprisingly, the CD10^+ ^LNCaP, C4-2, and LuCaP 35 were positioned near each other; LuCaP 35 and LNCaP were not exactly alike. LuCaP 49, being a neuroendocrine tumor type, was quite unlike any other in PCA. The G4 was also unlike any other. For the primary tumor cells, G3 was plotted closer to luminal L than G4, not unexpected since tumors with G4 cells do not display a glandular morphology. The small separation for G3 and L amounted to ~200 genes with > 8-fold difference in expression between the two [8]. The G3 cancer could be described as luminal-like. Luminal-like is here intended to mean that the cancer transcriptome is like that of luminal cells, i.e., luminal and G3 cells share many genes. In contrast, G4 and luminal cells share far fewer genes.

**Table 1:** Cancer cell CD phenotyping.

Cell type	CD57	CD44	CD107b	CD10	CD133	CD26
Luminal	+	-	+	+	-	+

Basal	-	+	-/+	-	-	-

5D3	-	+	-/+	-	+	-

G3	+	-	+	-	-	+

G4	+	-	+	-	-	+

LNCaP	-	-	+	+	-	-

C4-2	-	-	+	+	-	+

CL1	-	+	+	-	-	+

DU145	-	+	+	-	-	-

PC3	-	+	+	-	-	+

LuCaP35	-	-	+	+	-	-

LuCaP49	+	-	+	-	+	-

Non-luminal-like types are all representatives of aggressive disease, and this could be attributed to the similarity in overall gene expression to stem and progenitor cell types.

In PCA, a subgrouping can be seen for G3, LNCaP, C4-2 and LuCaP 35 about L (Figure 4B, left panel). Unlike the other three, the G3 cancer was immunotyped as CD10 negative (luminal is positive) [8]. Again, this analysis did not account for the genes involved in cellular proliferation, an important difference between sorted cells and cultured cell. A second subgrouping contained G4, CL1, DU145, PC3 and LuCaP 49 (Figure 4B, right panel). The 5D3 progenitor cell type could also be included in it. This subgrouping was positioned closer to the stem cell domain with CL1, PC3 and DU145 occupying a sub-domain. One may consider that G4 cells could represent a less differentiated cell type (i.e., closer to stem/progenitor) in the luminal epithelial lineage than G3 cells. These less luminal-like more stem-like cancer cell types are generally considered to have a higher malignant potential and represent advanced diseases. Outside some shared CD molecules, their overall gene expression was quite dissimilar to basal cells as well. The two groupings could be labeled as luminal-like and non-luminal-like (Table 2).

**Table 2:** Prostate cells, stem cells, cancer cell lines and xenografts.

Cell type	Origin	Characteristics	AR status	PSA/KLK3 status
***Prostate cancer cell lines***				

LNCaP	Lymph node metastasis	Luminal	Mutant AR	Positive
C4-2	Derived from LNCaP through selection in castrated animals	Luminal	Mutant AR	Positive
CL1	Derived from LNCaP through selection in androgen-depleted media	Non-luminal-like	Negative	Negative
PC3	Bone metastasis	Non-luminal-like	Negative	Negative
DU145	Brain metastasis	Non-luminal-like	Negative	Negative

***Xenografts***				

LuCaP 35	Lymph node metastasis, adenocarcinoma	Luminal-like	Positive	Positive
LuCaP 49	Omental metastasis, small cell carcinoma	Non-luminal-like	Negative	Negative

***Cells isolated from prostate tumor specimens***				

G3	CD26^+ ^Gleason pattern 3 from primary tumor, glandular	Luminal-like	Positive	Positive
G4	CD26^+ ^Gleason pattern 4 from primary tumor, aglandular	Non-luminal-like	Positive	Positive

***Stem cell lines***				

NCCIT	Embryonal carcinoma		Negative	Negative
H1	Embryonic stem cell		Negative	Negative

***Cells isolated from normal prostate tissue specimens***				

Luminal (L)	CD26^+ ^luminal epithelial		Positive	Positive
Basal (B)	CD104^+ ^basal epithelial		Negative	Negative
Stromal (S)	CD49a^+ ^stromal		Positive	Negative
Endothelial (E)	CD31^+ ^endothelial		Negative	Negative
Progenitor (5D3)	ABCG2^+ ^progenitor		Negative	Negative
Progenitor (SP)	Side population progenitor		Negative	Negative

### 3. Candidate prostate cancer stem cells

**Figure 5:**
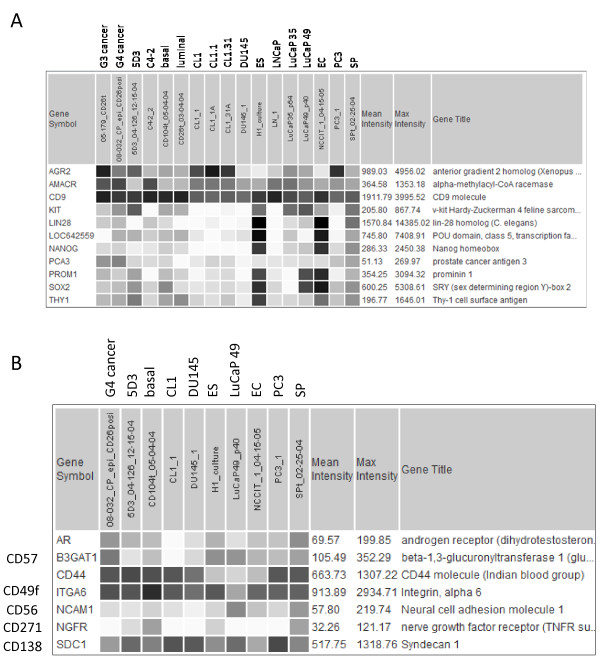
**Differential gene expression among cancer cell types**. A: Shown is the dataset query display of selected genes among the cell types listed. B: Shown is the query display for seven selected genes among the non-luminal-like cancer cell types.

Like 5D3 and SP cells, the different non-luminal-like prostate cancer cell types showed little expression of LIN28, POU5F1, NANOG, SOX2 (Figure 5A). This was not unexpected for the luminal-like types. The cancer cells (except LuCaP49) could be distinguished by their expression of any one of the cancer genes AMACR, PCA3, AGR2; their expression levels were variable. LuCaP 49 had signals for KIT (CD117) and SOX2 in addition to PROM1. Both 5D3 and SP also showed expression of KIT. A single cell type with this marker was reported to be capable of generating a prostate [33]. Figure 5B shows the expression pattern of several other genes in these cancer cell types. Except for G4, AR expression was minimal in the other non-luminal-like types and the progenitor cells. CD44, CD49f, CD271, CD138 are basal cell markers, and some could be detected in the cancer cells. Note the lower expression of CD44, by comparison, in the ES and EC cells. LuCaP 49 showed markers of neuroendocrine differentiation CD56 and CD57, and was unique in that regard.

**Figure 6:**
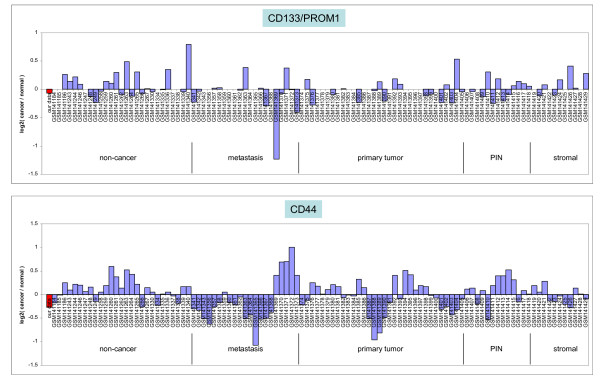
**Prostate tumor CD44 and CD133 expression**. Dataset identities in the histograms are indicated on the *x*-axis. Red is that of 05-179 CD26^+ ^G3 cancer cells, which shows absent CD44 and CD133 expression.

To gauge the frequency of CD44-positive or CD133-positive prostate tumors, a large publicly available prostate cancer dataset [34] was queried. The CD44^-^CD133^- ^G3 cancer transcriptome was included for comparison (Figure 6). As can be seen, cancer expression of CD133 in these laser-capture microdissected tumor cell specimens was very infrequent. There was more cancer CD44 expression in both primary tumors and metastases. Low frequencies of tumors positive for CD133 (< 1% primary, < 4% bone metastasis) and CD44 (< 10% metastasis) by immunostaining were recently reported by Eaton *et al*. [35]. CD44 and CD133 expression detected in non-cancer could be due to basal or other cell types as total prostate RNA was used as non-cancer in that analysis [34].

## Discussion

Transcriptome comparison between prostate cancer cells isolated from a Gleason 3+3 tumor and normal cell types has shown relatedness between CD26^+ ^cancer and CD26^+ ^luminal cells [8]. Gleason pattern 3 cells are thought to represent the disease at an early, well-differentiated, stage. Several possibilities could account for the luminal-like cancer phenotype. One, cancer cells may originate from a human equivalent of the so-called bipotent castration-resistant Nkx3.1-positive mouse luminal cell type (CARN) upon loss of Pten function [36]. Two, cancer cells may arise from a human equivalent of the Cd49f-positive mouse basal cell type under the influence of activated Akt, Fgf10, Erg1 and Ar. The resultant cancer cells would show a luminal phenotype as basal cells are the progenitors of secretory cells [37]. Three, cancer cells may result from luminal differentiation of a neoplastic progenitor-like cell type containing genetic alterations such as the TMPRSS2:ERG fusion [38]. ERG1 was one of the genes with increased expression in this G3 cancer cell type [8], and its expression could result from a gene fusion event placing it under the control of the androgen regulated TMPRSS2 [39]. This tumor-initiating cell type could, as postulated, both renew and propagate tumor formation. These models and others (e.g., the TRAMP mouse engineered with an activatable SV40 T antigen [40]) of prostate carcinogenesis invariably involve oncogene activation. An alternative to this involves cell-cell signaling. Cancer differentiation takes place because tumor-associated stromal cells are functionally defective due to their down-regulated expression of certain organ-restricted genes and genes in smooth muscle cell differentiation [7]. Stromal cells in normal tissue are characterized as smooth muscle cells. Organ-specific stromal induction of epithelial differentiation could be attributed to the genes differentially expressed between prostate and, say, bladder stromal cells. Stem/progenitor cells differentiate in response to the cues provided by the stromal elements. In this model, a prostate cancer stem cell type is not required. As these cues from tumor-associated stromal cells are different, functional luminal differentiation cannot be achieved resulting, for example, in the absent expression of luminal CD10 and CD13 [8]. These membrane peptidase enzymes are likely important in processing signaling protein/peptide molecules. Furthermore, depending on the extent of stromal defect, different cancer cell types, e.g., G3 or G4, may arise. Our study with co-cultures of tumor-associated stromal cells and NCCIT has shown difference in the induced gene expression of treated NCCIT cells by these stromal cells compared to that by normal tissue stromal cells [41].

Compared to the luminal-like types, the non-luminal-like cancer cells show less features of secretory differentiation such as decreased expression of KLK3 and AR, and expression of markers associated with more primitive cell types. This is supported by the "migration" of their transcriptomes away from luminal towards stem/progenitor. Some have gained perhaps certain functional properties of stem cells, which was reported for PC3 [42] and DU145 [43]. Whether they would show response to stromal induction is yet to be demonstrated with full transcriptome analysis. As hormone influence plays an important role in prostate cytodifferentiation [5], lack of androgen may also lead to increased expression of progenitor cell markers (CD133, CD44, CD117, ABCG2) in cancer cells [44]. However, based on our data analysis and data reported in the literature, stem cell marker expression in prostate cancer cells appears haphazard. Some markers, for example, like CD49f/ITGA6 could be detected frequently in metastases while others like nestin could not [35]. Thus, there is no hard evidence for the existence of a cancer stem cell type with gene expression similar to ES (or EC) cells in prostate cancer. Based on overall gene expression, the closest appears to be the trio of PC3, DU145 and CL1.

In breast cancer, a luminal-type is also known, and a basal-type has been described to be more aggressive than the luminal-type [45]. Although a number of basal CD molecules are found, none of the prostate cancer cell types show similarity to basal cells in gene expression. Basal cells, and not luminal cells, can be cultured and transformed into immortalized cell lines. Many experiments have used these cells as a model for normal prostate epithelial cells. They can be induced by carcinoma-associated fibroblasts (CAF) to produce highly malignant progeny (i.e., the non-luminal type) [46]. The large transcriptome difference between basal cells and stem cells as shown by PCA suggests that basal cells are not likely to possess functional properties of stem cells. Perhaps, the expression signature of basal-derived cancer cells would turn out to be distinct from the ones analyzed here.

If a cancer stem cell is not necessarily needed for tumor development, then can a normal prostate stem cell be programmed to produce tumor cells? The existence of a stem cell population in the prostate was inferred from animal studies, in which castration leads to involution of the gland, and hormone administration produces recovery [47]. Androgen removal has a deleterious effect on the AR-positive luminal cells but a minimal one on the AR-negative basal cells. Progenitor cells that could repopulate the gland might therefore reside in the basal epithelium. Work on the epidermis estimated the stem cell population to be 10% of cells in the basal layer [48]. About 3% of the basal cells show rapid adhesion to type I collagen and have a 4-fold higher colony forming efficiency than non-adherent cells. The adherent cells also form glands in a mouse host when co-transplanted with human stromal cells [49]. The ABCG2 population as represented by the 04-126 sort could be the prostate progenitor. These ABCG2^+^CD31^-^CD138^+ ^(and CD117^+^) cells were estimated to comprise about < 1% of the cells in the basal epithelium. The SP cells showed a close gene expression to the ABGC2^+ ^cells, and were negative for AR expression [18]. Therefore, it is possible that this cell type could give rise to either normal or cancer cell types depending on the stromal signaling. We are trying to determine if a cell line can be established from cells isolated by the 5D3 antibody. The technical challenge is the frequent co-isolation of endothelial cells.

## Conclusions

Transcriptomics can identify luminal-like and non-luminal-like prostate cancer cell types. A putative progenitor cell population could be isolated by methods based on the expression of ABCG2. The ABCG2^+ ^cells and the non-luminal-like cancer cells are more similar to stem cells in overall gene expression than the luminal-like cancer cells. Although basal cells have been postulated to be the progenitors of luminal cells and used as a model of normal epithelial cells in cancer development studies, none of the cancer cell types show a basal-like gene expression signature.

## Abbreviations

ABCG2: ATP-binding cassette, sub-family G (white), member 2; AMACR: α-methylacyl-CoA racemase; AGR2: anterior gradient 2; AR: androgen receptor; B: prostate basal epithelial cells; B3GAT1 (CD57): β-1,3-glucuronyltransferase 1; CD: cluster designation; CD26/DPP4: dipeptidyl peptidase 4; E: endothelial cells of blood capillaries; EC: embryonal carcinoma; ERG1: early growth response 1; ES: embryonic stem; EST: expressed sequence tag; G3: Gleason pattern 3; G4: Gleason pattern 4; ITGA6/CD49f: integrin α6; KIT/CD117: *v*-kit Hardy-Zuckerman 4 feline sarcoma viral oncogene homolog; L: prostate luminal epithelial cells; LAMP2/CD107b: lysosomal-associated membrane protein 2; LIN28: lin-28 homolog (*C*. *elegans*); MACS: magnetic cell sorting; MME/CD10: membrane metallo-endopeptidase; NANOG: Nanog homeobox; NCAM1/CD56: neural cell adhesion molecule 1; NGFR/CD271: nerve growth factor receptor: PCA: principal components analysis; PCA3: prostate cancer antigen 3 (non-protein coding); PECAM1/CD31: platelet/endothelial cell adhesion molecule; POU5F1/OCT4: POU class 5 homeobox 1; PROM1/CD133: prominin 1; PSA/KLK3: prostate-specific antigen/kallikrein-related peptidase 3; S: prostate stromal smooth muscle cells; SDC1/CD138: syndecan 1; SOX2: SRY (sex determining Y)-box 2; SP: side population; TDGF1: teratocarcinoma-derived growth factor 1; THY1/CD90: thymus cell antigen 1; TMPRSS2: transmembrane protease, serine 2; TRAMP: transgenic adenocarcinoma of the mouse prostate.

## Competing interests

The authors declare that they have no competing interests.

## Authors' contributions

LEP and AYL designed research; LEP, AYL performed research; LEP, RZNV, EFV, GD and AYL analyzed data; RLV provided the xenografts; CBW provided the ES cells; LEP and AYL wrote the manuscript with contribution from the coauthors. All authors read and approved the final manuscript.

## Pre-publication history

The pre-publication history for this paper can be accessed here:

http://www.biomedcentral.com/1755-8794/4/46/prepub
